# Humeral head replacement for syringomyelia-associated Charcot shoulder arthropathy: a case report and literature review

**DOI:** 10.3389/fsurg.2026.1741848

**Published:** 2026-04-08

**Authors:** Lin Zhang, Jinglin Li, Fuyin Yang, Jiaze Peng, Yang Yu, Xianpeng Huang, Xuan Deng, Xuxu Yang, Lidan Yang

**Affiliations:** Department of Orthopedics, Affiliated Hospital of Zunyi Medical University, Zunyi, China

**Keywords:** case report, Charcot arthropathy, humeral head replacement, literature review, syringomyelia

## Abstract

**Background:**

Syringomyelia combined with Charcot shoulder arthropathy is a highly disabling neuropathic joint disorder. The core pathology involves dual damage to pain/temperature sensation and sympathetic fibers due to the syrinx, leading the glenohumeral joint to endure abnormal mechanical stress without neural protection, resulting in bone and joint destruction. However, due to the rarity of the disease, its treatment is highly controversial. This paper reports a case of humeral head replacement for Charcot shoulder arthropathy caused by syringomyelia and reviews the relevant literature for reference.

**Case presentation:**

A 50-year-old female patient presented to our hospital with right shoulder pain and limited mobility for 6 years. Examination revealed syringomyelia combined with right shoulder Charcot arthropathy and right shoulder dislocation. We addressed the patient's joint pathology using humeral head replacement and rotator cuff repair; her syringomyelia and Chiari malformation were scheduled for secondary surgery. At the one-year postoperative follow-up, numbness on the dorsum of the right hand was significantly reduced, the range of motion of the right shoulder joint was markedly increased, and the shoulder Constant–Murley score was 80.

**Conclusion:**

Performing joint replacement first for secondary Charcot arthropathy to alleviate pain and meet the patient's functional needs, followed by elective surgery for the primary neurological disease, may be more suitable for patients with syringomyelia combined with end-stage Charcot arthropathy. For such patients, multidisciplinary collaboration and long-term follow-up are essential to optimize surgical treatment strategies and improve long-term outcomes.

## Background

Syringomyelia is a chronic, progressive neurological disease characterized by cystic cavitation within the spinal cord. This lesion destroys pain/temperature sensation and sympathetic fibers within the cord, leading to protective sensory deficits in corresponding dermatomes and neurotrophic imbalance, thereby inducing neuropathic arthropathy (1). The shoulder joint is one of the most commonly affected sites, manifesting as recurrent occult trauma, rapidly progressive osteolysis, joint collapse, and instability, ultimately resulting in complete joint destruction and severe functional impairment (1). Due to the extreme rarity of syringomyelia combined with Charcot shoulder secondary to syringomyelia (CSSS), there is a lack of evidence-based medical evidence and consensus guidelines, posing significant challenges for clinical decision-making. Traditional conservative treatments have unclear efficacy in halting joint destruction, while joint arthrodesis has gradually been phased out due to significant functional loss. Although total shoulder or reverse shoulder arthroplasty can restore some range of motion, neuropathic bone defects, rotator cuff/ligament insufficiency, and persistent abnormal stress significantly increase the risk of prosthesis loosening, infection, and fracture, resulting in a high postoperative failure rate (2, 3). Humeral head replacement (HHR), which preserves glenoid bone stock and involves relatively simplified procedures, is theoretically considered to reduce complications; however, its feasibility, surgical technique, and early functional outcomes in CSSS have only been sporadically reported in case studies. Furthermore, CSSS patients often initially present with joint symptoms, leading to delayed diagnosis and treatment of the primary neurological disorder, resulting in irreversible nerve damage and joint destruction. Therefore, clarifying surgical strategies for end-stage CSSS, optimizing multidisciplinary collaboration pathways, and evaluating long-term efficacy are of urgent clinical significance for improving patient outcomes. This study reports a case of CSSS managed by a multidisciplinary team (MDT) through initial HHR combined with rotator cuff repair to reconstruct glenohumeral joint function, with planned secondary management of Chiari malformation and syringomyelia, and reviews relevant literature to provide insights into the treatment of CSSS.

## Case presentation

### Preoperative

A 50-year-old right-handed female patient. She began experiencing dull pain in the right shoulder and limited shoulder mobility 6 years prior, which gradually worsened, affecting combing hair and dressing; she later developed numbness over the dorsum of the right hand in the area of the 1st to 3rd metacarpals. She engaged in light physical labor, had no history of diabetes, smoking, steroid use or alcohol abuse, and no history of infection, trauma, or surgery in the right shoulder joint. Physical examination revealed no significant redness, hotness or swelling in the right shoulder, slight tenderness, right shoulder range of motion: forward flexion 90°, extension 20°, abduction 90°, adduction 20°, internal and external rotation 10°. The right shoulder and bilateral elbow joints were stable, pain-free, and demonstrated normal range of motion.

**Figure 1:**
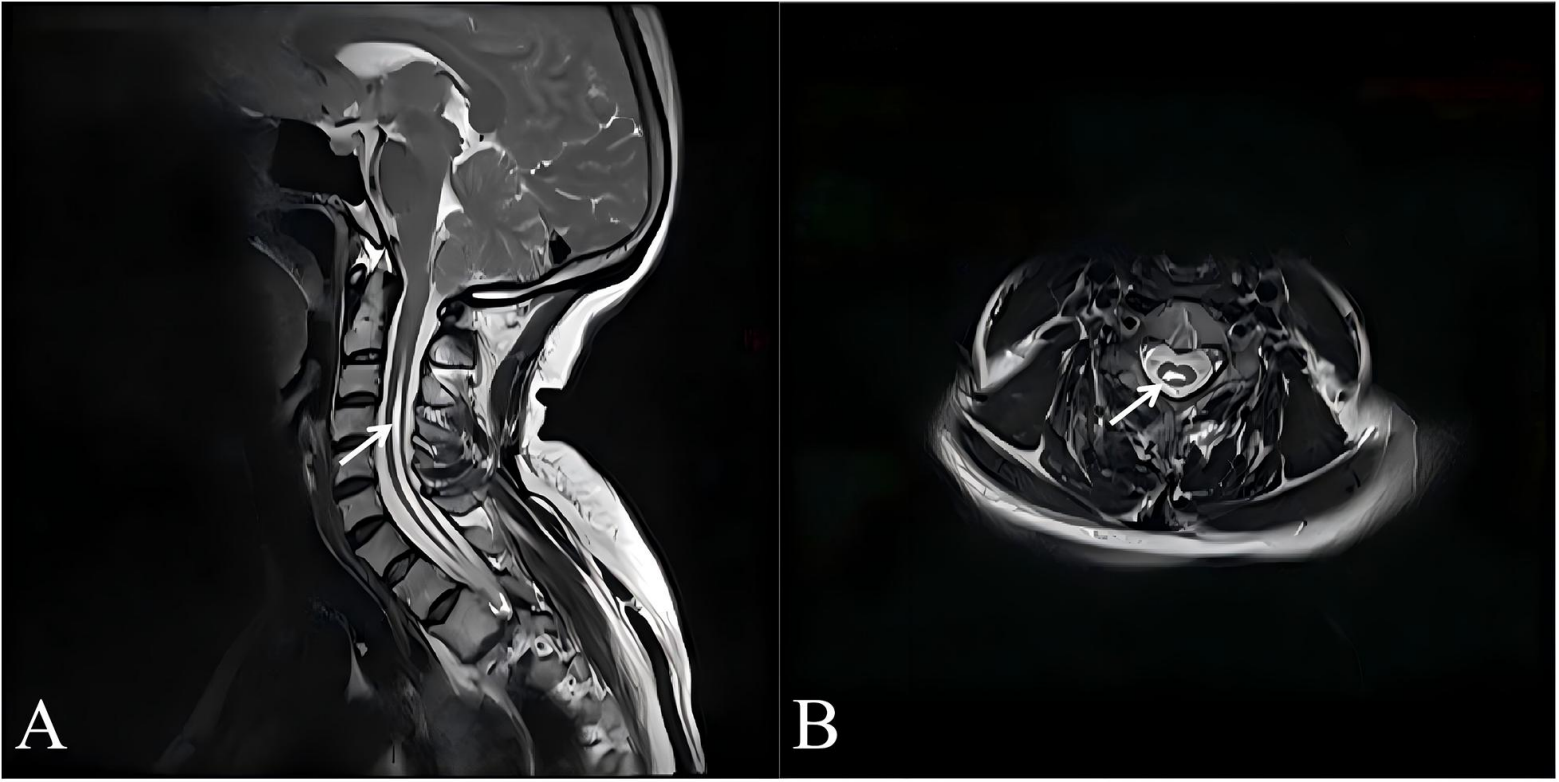
Preoperative cervical spine MRI. Sagittal view **(A)** and axial view **(B)**. The white arrows in **A** and **B** indicate the syrinx cavity.

**Figure 2:**
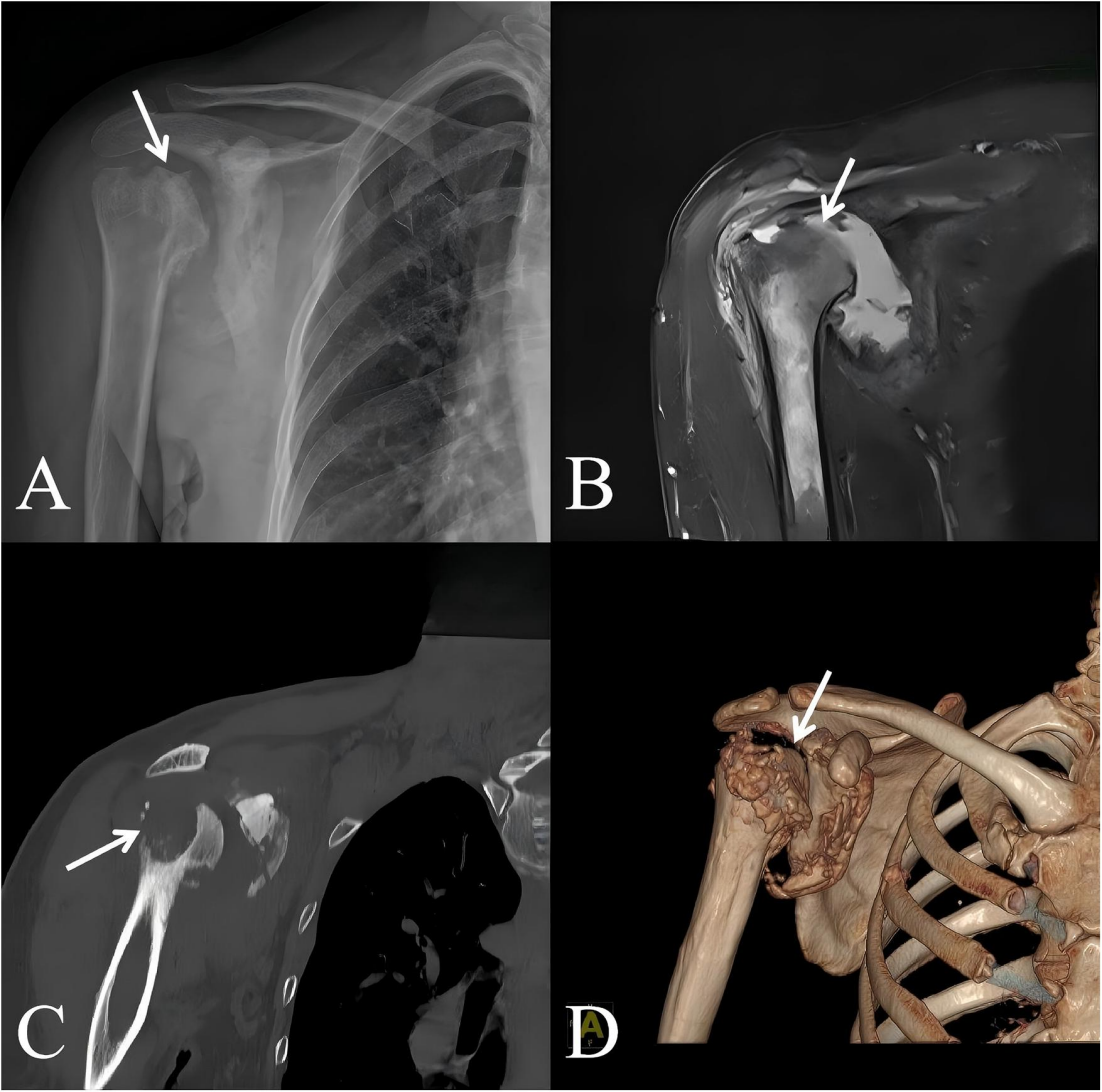
Preoperative right shoulder anteroposterior X-ray **(A)**, MRI coronal view **(B)**, CT coronal view **(C)**, and 3D reconstruction **(D)**. The white arrows in **A–D** indicate the osteolytic destruction of the humeral head.

Neurological examination revealed decreased pain and temperature sensation over the dorsum of the right hand, mainly involving the C6–C7 dermatomal distribution. Light touch sensation was relatively preserved. Despite the sensory deficits, Motor strength of the bilateral upper extremities was normal, with no muscle atrophy observed. Biceps and triceps reflexes were normal, suggesting that the syrinx predominantly affected the spinothalamic tract while sparing the ventral horn and reflex arcs, a finding consistent with the dissociated sensory loss characteristic of syringomyelia. Laboratory tests: CRP, ESR normal; Glycated hemoglobin 5.2%. Cervical MRI: Syringomyelia from C2-T5, maximum width 7 mm, cerebellar tonsillar herniation 8 mm (Figures 1A, B). Right shoulder X-ray and CT: Osteolytic bone destruction of the joint components, right shoulder dislocation (Figures 2A–D); MRI showed disappearance of the right glenoid structure, large defect area in the humeral head. The right shoulder joint capsule was enlarged, with large areas of edema in the periarticular soft tissues (Figure 2B). Although avascular necrosis was considered in the differential diagnosis, it was ruled out due to the absence of typical risk factors and imaging features. MRI revealed no subchondral crescent sign or demarcated infarcted area. Instead, diffuse osteolysis, joint effusion, and periarticular soft tissue edema were observed, findings more consistent with neuropathic arthropathy. Therefore, the final diagnosis is Syringomyelia with chiari malformation type I, right shoulder charcot arthropathy and right shoulder dislocation.

### Surgical management

After multidisciplinary discussion, right shoulder humeral head replacement was performed first. After anesthesia, the patient was placed in a beach-chair position, routinely sterilized and draped. A deltopectoral approach was used, starting from the coracoid process, approximately 10 cm long. The skin, subcutaneous tissue, and superficial fascia were incised layer by layer with an electrocautery. The deltoid was split along its anterior border, with a few muscle fibers and the cephalic vein retracted medially, exposing the rotator cuff, joint capsule, and the insertion of the pectoralis major. The long head of the biceps tendon was found ruptured and retracted, and was marked. The joint capsule was incised, and the subscapularis tendon along with the capsule was retracted anteriorly. Exploration revealed most of the greater tuberosity had dissolved, part of the lesser tuberosity remained, the anterior half and inferior two-thirds of the humeral head were largely dissolved, and the posterior half had partial loss of cartilage and subchondral bone. After positioning, the residual humeral head was resected using a bone micro-oscillating saw. The proximal humeral medullary canal was reamed. A No. 2 stem trial was installed referencing the bicipital groove, and the height was determined based on the pectoralis major tendon insertion. Granulation tissue and bone fragments within the joint were debrided. A Lima SMR size 50 CTA humeral head trial was used, and fluoroscopy confirmed appropriate positioning. The trial was replaced with the corresponding size prosthesis. Checked range of motion in all directions was good, with no dislocation. The subscapularis tendon was repaired and sutured near the original greater tuberosity site using sutures, and the long head of the biceps tendon was sutured locally. Shoulder range of motion was checked again and found to be good. End of surgery. The cerebellar tonsillar herniation and syringomyelia had indications for surgery, and occipitocervical decompression was planned electively.

Six months after the humeral head replacement surgery, the patient underwent occipitocervical decompression and partial resection of the cerebellar tonsils. After the anesthesia took effect, the patient was placed in the left lateral decubitus position. Following routine disinfection and draping, a midline suboccipital craniotomy approach was adopted. An approximately 6 cm incision was made from the external occipital protuberance to the superior border of the C2 vertebra. Layers were dissected to expose the foramen magnum region, the posterior arch of C1, and the spinous process of C2. The posterior rim and both lateral aspects of the foramen magnum were removed, creating a bone window of approximately 1.5 × 3 cm for decompression. Concurrently, thickened atlanto-occipital membrane was excised using a rongeur to achieve adequate spinal canal decompression. The posterior arch of the atlas was removed to a width of approximately 3 cm. The occipital sinus and marginal sinus were found to be well-developed and were incised linearly and ligated. The cerebellar tonsils were observed to be displaced downward to the level of the superior border of C2, with adhesions present between the inferior cerebellar surface/cerebellar tonsils and the dura mater. The cisterna magna was obliterated, and the ventral aspects of the cerebellar tonsils were adherent to the cervicomedullary junction. Partial resection of the cerebellar tonsils was performed up to the level above the foramen magnum. Exploration confirmed satisfactory exposure of the cervical nerve roots. Further exploration verified a patent communication between the central canal of the spinal cord and the foramen of Magendie, with clear, colorless cerebrospinal fluid flowing freely. An artificial dura mater patch was secured with 5–0 sutures for watertight dural closure. The epidural space was covered with gelatin sponge and a large biological sheet. A drainage tube was placed in the surgical site. Closure was performed in layers, concluding the procedure.

### Postoperative outcome

**Figure 3:**
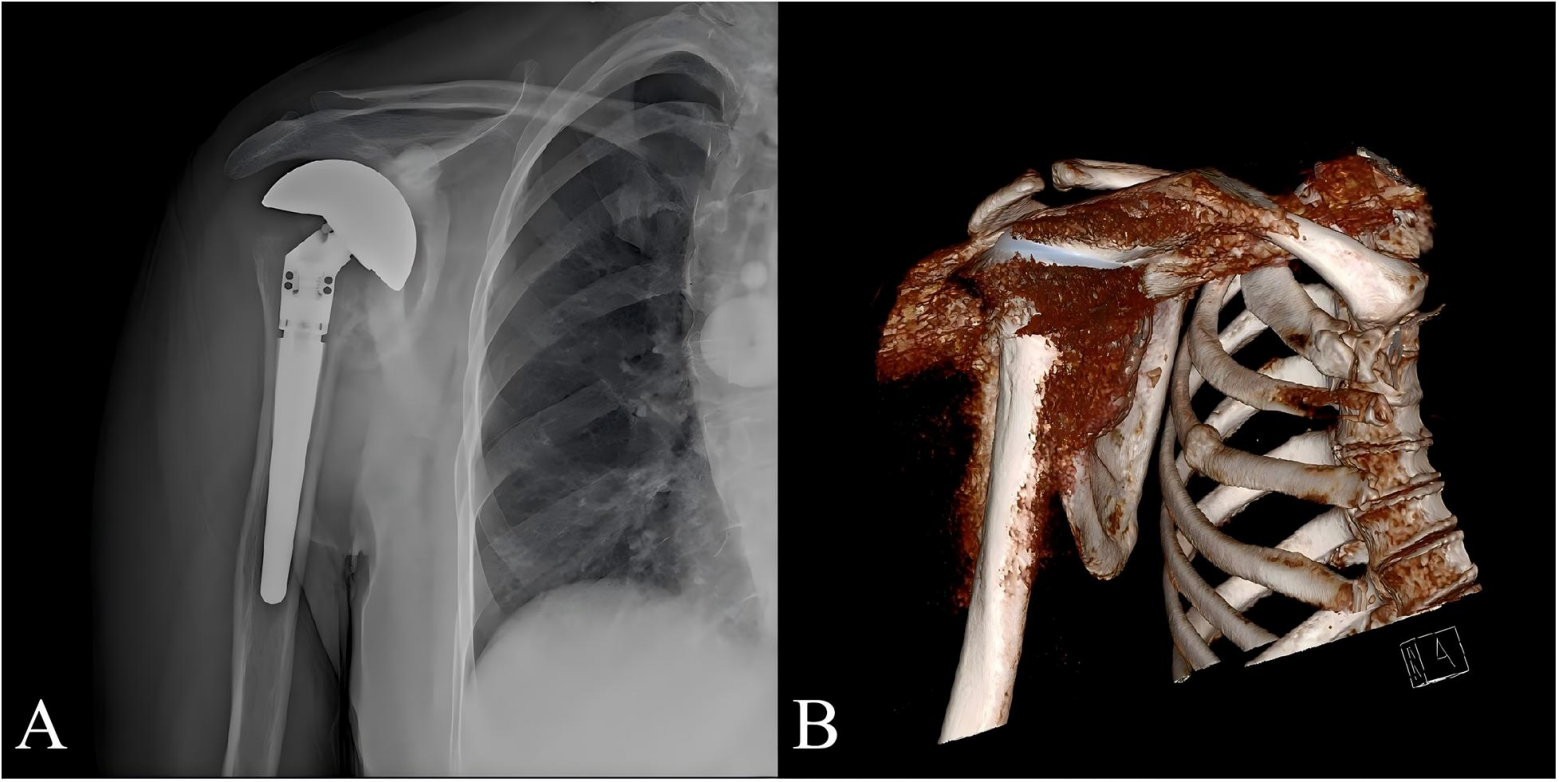
One-year postoperative anteroposterior shoulder X-ray **(A)** and 3D reconstruction **(B)** showing the prosthesis securely in place.

At 6 weeks after shoulder surgery, the patient was instructed to gradually begin active exercises for internal rotation, forward flexion, and abduction of the shoulder joint. Strength training began at 8 weeks postoperatively. Six months after humeral head replacement, the patient demonstrated a marked improvement in shoulder range of motion compared with the preoperative status. At the 1-year follow-up after neurosurgery, the prosthesis was stable and in place (Figures 3A, B), and the patient could perform normal physical activities, and numbness on the dorsum of the right hand was significantly reduced. Physical examination of the right shoulder range of motion was significantly increased: forward flexion 150°, extension 45°, abduction 150°, adduction 45°, internal rotation 45°, external rotation 90°. Contralateral shoulder and bilateral elbow joints revealed stability, absence of pain, and normal range of motion. Imaging studies showed no significant pathological changes. The right shoulder Constant–Murley score was 80. Given that the underlying neurological condition may persist or only partially improve, long-term management is essential. Our plan includes annual clinical and radiographic follow-up to assess prosthesis integrity and detect early signs of loosening or infection. Neurological re-evaluation with MRI is indicated if new symptoms arise. The patient was advised to protect joint and modify activity to maintain range of motion and muscle strength without overloading the join.

## Discussion

Charcot shoulder arthropathy is a condition that occurs in patients with neurological diseases, progresses slowly, and causes joint damage affecting activities of daily living. One of its main causes is syringomyelia in the cervical region (4). Syringomyelia combined with Charcot shoulder secondary to syringomyelia (CSSS) is a rare and highly disabling neuropathic joint disorder. Its core pathology involves dual damage to pain/temperature sensation and sympathetic fibers due to the syrinx, leading the glenohumeral joint to endure abnormal mechanical stress without neural protection, eventually resulting in bone fragmentation and joint dislocation (5). The 50-year-old female patient in this case, with right shoulder pain and dysfunction lasting 6 years, imaging showing significant humeral head dissolution and shoulder dislocation, accompanied by C2-T5 segment syringomyelia and Chiari Malformation Type I, represents a typical presentation of end-stage CSSS.

The treatment goals for patients with neuropathic arthropathy (NA) are early diagnosis, maintenance of joint and limb function, and treatment of the underlying disease. However, there are no consensus guidelines in the current literature for the treatment of NA related to syringomyelia. Treatment modalities range from conservative approaches such as rehabilitation, joint protection strategies, education, and analgesics, to arthrodesis, prosthetic replacement, and neurosurgical intervention (6, 7). Treatment of CSSS needs to address both the primary neurological disease and the secondary joint pathology. According to the literature, surgical intervention is considered a more rational treatment option for reducing pain and improving functional status in CSSS patients (2).

For the treatment of the primary disease, the main goal of intervention is to prevent further expansion of the syrinx and avoid damage to the remaining spinal cord areas. Existing data suggest that early treatment of the primary neurological disease may halt the development of Charcot arthropathy. Deng et al. (8), in a study of 12 patients with Charcot arthropathy caused by syringomyelia, found that 5 patients who underwent neurosurgical treatment for the primary disease showed improved neurological deficits postoperatively, and the related joint symptoms did not worsen. Among the 7 patients who did not undergo surgery, 5 experienced progression of joint destruction and neurological deterioration. Makihara et al. (9) reviewed a patient with Charcot shoulder who underwent suboccipital decompression for syringomyelia, showing improved proximal muscle strength and reduced sensory changes. Over time, bone regeneration resembling a new joint surface occurred. Atalar et al. (10) reviewed 5 patients with syringomyelia and neuropathic shoulder joints; all patients who underwent neurosurgical decompression showed improvement in the range of motion and function of the neuropathic joint.

However, most patients present initially with shoulder problems, while the primary neurological disease remains undiagnosed, often leading to significant delays in diagnosis and treatment, and ultimately resulting in irreversible end-stage Charcot shoulder that is difficult to manage (11). In end-stage Charcot shoulder secondary to syringomyelia, the main pathological driver of the joint shifts from chronic nerve damage to acute mechanical instability. This stage involves extensive bone defects, joint surface collapse, and large-scale destruction of soft tissues such as tendons. Abnormal geometry and soft tissue imbalance lead to significantly increased shear forces and point stress, forming a continuous cycle of bone resorption and re-destruction. Although spinal decompression surgery can halt syrinx expansion, neurological recovery is typically slow and incomplete. Heiss et al. reported that the incidence of recurrent or residual syringomyelia after decompressive surgery is 10%–40% (12). In other words, persistent or recurrent syrinx can remain post-decompression, indicating that neurosurgical intervention may not achieve sufficient functional recovery in all cases. Furthermore, Arabzadeh et al. showed that shoulder Charcot arthropathy can progress even after the primary neurological cause has been addressed via decompression (13). In summary, neurological and functional recovery after decompression often takes months to years and may be limited in some patients, making it difficult to reverse existing structural damage to bone and soft tissues within the joint in the short term. In contrast, joint replacement and soft tissue reconstruction can immediately restore force couples and load distribution, reduce abnormal stress concentration, halt further bone destruction, and provide a stable biomechanical foundation for rehabilitation. Therefore, prioritizing orthopedic reconstruction in the end-stage structural destruction phase, followed by neurosurgical decompression to control the primary etiology, represents a reasonable strategy balancing immediate stability and long-term control. This patient's Chiari malformation and syringomyelia had clear indications for neurosurgery, but the MDT decided to perform HHR first, followed by staged occipitocervical decompression, primarily based on: 1) The joint was already dislocated with massive bone defects, and delayed reconstruction would further increase glenoid and proximal humeral bone loss; 2) Neurological symptom progression was relatively slow (only numbness on the dorsum of the hand), suggesting a controllable short-term risk of syrinx expansion; 3) Stabilizing the shoulder first could provide a tolerable basis for early limb movement rehabilitation after the second-stage suboccipital decompression. This “reverse sequential” strategy reflects a patient functional needs-oriented, individualized MDT decision-making process.

For the treatment of secondary neuropathic shoulder arthropathy, options include shoulder arthrodesis, shoulder resurfacing arthroplasty, reverse shoulder arthroplasty (RSA), and humeral head replacement (HHR). Arthrodesis has been gradually abandoned due to significant functional loss and suboptimal outcomes (14, 15). Shoulder resurfacing arthroplasty preserves existing humeral head and glenoid bone stock for future surgeries; this procedure is suitable for young neuropathic shoulder patients with sufficient humeral head bone stock (16). The recently popularized reverse shoulder arthroplasty (RSA) is suitable for patients after conservative measures have failed, with a rounded glenoid, humeral head collapse, massive irreparable rotator cuff tear, but with an intact deltoid muscle (17). However, serious complications such as postoperative infection, periprosthetic fracture, and prosthesis loosening make this procedure highly controversial. Schoch et al. from the USA (18) reported 10 patients who underwent shoulder arthroplasty after failed conservative treatment for Charcot shoulder; among the 3 cases that underwent RSA, 2 developed acromial stress fractures during follow-up. Some scholars also note that Charcot shoulder, due to poor bone quality, prosthesis loosening, instability, and soft tissue damage, is a contraindication for total shoulder arthroplasty (19). Some literature shows that humeral head replacement (HHR) has achieved satisfactory outcomes when combined with rotator cuff preservation or reconstruction (4, 20). Matsuhashi et al. (20) performed HHR and rotator cuff repair on 3 neuropathic shoulder joints following initial neurosurgical decompression of the syrinx cavity. After 8 years, patients had reduced pain, increased range of motion, and no signs of prosthesis loosening. By performing rotator cuff repair along with arthroplasty, we believe this provides better head stability, thereby reducing complication rates. In this case, the intraoperative findings included severe changes such as extensive dissolution of the humeral head, greater tuberosity defect, and rupture of the long head of the biceps tendon. Stability was achieved through simple HHR combined with reconstruction of the subscapularis tendon to the original greater tuberosity attachment site using 4 sutures. The 1-year follow-up showed significant improvement in shoulder range of motion, a Constant–Murley score of 80, and reduced numbness on the dorsum of the right hand, indicating that even without addressing the primary neurological lesion, joint replacement can provide significant symptomatic relief and functional improvement.

Despite the favorable clinical and functional outcomes observed in this patient, several limitations of the present study should be acknowledged. On the one hand, this report describes a single-case experience, which inherently limits the generalizability of the findings and precludes definitive conclusions regarding the optimal treatment sequence for syringomyelia-associated Charcot shoulder arthropathy. Individual variability in neurological status, extent of joint destruction, and soft-tissue integrity may substantially influence surgical decision-making and outcomes. On the other hand, although the patient demonstrated satisfactory short- to mid-term results following staged orthopedic reconstruction and subsequent neurosurgical decompression, the duration of follow-up remains limited. Longer-term observation is necessary to evaluate prosthesis survivorship, the risk of late loosening, instability, or periprosthetic fracture, as well as the long-term interaction between joint reconstruction and control of the underlying neurological disease. Therefore, larger case series, multicenter studies, and extended follow-up are required to validate the proposed staged treatment strategy and to establish more robust clinical recommendations for this rare and challenging condition.

## Conclusion

Performing joint replacement first for secondary Charcot arthropathy to alleviate pain and meet the patient's functional needs, followed by elective surgery for the primary neurological disease, may be more suitable for patients with syringomyelia combined with end-stage Charcot arthropathy. For such patients, multidisciplinary collaboration and long-term follow-up are essential to optimize surgical treatment strategies and improve long-term outcomes.

## Data Availability

The original contributions presented in the study are included in the article/Supplementary Material, further inquiries can be directed to the corresponding author.
